# Assessing the impact of multiple ultraviolet disinfection cycles on N95 filtering facepiece respirator integrity

**DOI:** 10.1038/s41598-021-91706-1

**Published:** 2021-06-10

**Authors:** C. Carolina Ontiveros, Crystal L. Sweeney, Christopher Smith, Sean MacIsaac, Jessica L. Bennett, Sebastian Munoz, Amina K. Stoddart, Graham A. Gagnon

**Affiliations:** 1 Department of Civil and Resource Engineering, Centre for Water Resources and Studies, Dalhousie University, 1360 Barrington St., Halifax, NS B3H 4R2 Canada; 2 Nova Scotia Health Authority, 90 Lovett Lake Court, Suite 201, Halifax, NS B3S 0H6 Canada

**Keywords:** Civil engineering, Health policy, Public health

## Abstract

During the COVID-19 pandemic, N95 filtering facepiece respirators (FFRs) were recommended to protect healthcare workers when providing care to infected patients. Despite their single-use disposable nature, the need to disinfect and repurpose FFRs is paramount during this global emergency. The objectives of this study were to (1) determine if UV treatment has an observable impact on respirator integrity; (2) test the impact of UV treatment on N95 FFR user fit; and (3) test the impact of UV treatment on FFR integrity. Ultraviolet (UV) disinfection was assessed in maintaining N95 FFR integrity. Two models of FFRs were exposed to UV fluences ranging from 0 to 10,000 mJ cm^−2^ per side and subsequently tested for fit, respirator integrity, and airflow. Inspection of N95 FFRs before and after UV treatment via microscopy methods showed no observable or tactile abnormalities in the integrity of respirator material or straps. Tensile loading tests on UV-treated and untreated respirator straps also demonstrated no impact on breaking strength. Standardized fit test methods showed no compromise in user fit following UV treatment. Evaluation of particle penetration and airflow through N95 FFRs showed no impact on integrity, and average filtration efficiency did not fall below 95% for any of the respirator types or fluence levels. This work provides evidence that UV disinfection does not compromise N95 FFR integrity at UV fluences up to 10,000 mJ cm^−2^. UV disinfection is a viable treatment option to support healthcare professionals in their strategy against the spread of COVID-19.

## Introduction

By the end of June 2020, the number of global cases of COVID-19, the illness caused by the novel severe acute respiratory syndrome coronavirus 2 (SARS-CoV-2), had surpassed 10 million and the death toll exceeded 500,000^1^. The transmission of COVID-19 occurs mainly through respiratory droplets from coughs and sneezes of infected individuals^2^. In order to mitigate disease transmission, the N95 filtering facepiece respirator (FFR) or higher-level respirator is recommended for performing aerosol-generating medical procedures (AGMP) in infected patients, such as open suctioning of airways, cardiopulmonary resuscitation, endotracheal intubation and extubation, and manual ventilation^3,4^. Amid this global emergency, the increasing demand for N95 FFRs is outpacing availability, and the development of timely methods for disinfecting and repurposing single-use FFRs is imperative.

The N95 FFR, approved by the National Institute for Occupational Safety and Health (NIOSH), is a single-use disposable respirator that filters out more than 95% of small particles in the air, including bacteria and viruses^4^. Respirator filtration is primarily governed by electrostatic removal (Online Resource ESM Fig. 1)^5^. As such, disinfection processes that cause changes in the surface charge render N95 rated respirators ineffective. Several methods for disinfecting respirators have been explored and resulted in a spectrum of outcomes in integrity. For example, microwave irradiation resulted in the melting of respirators to the point of being unwearable and untestable, while cleaning respirators with bleach resulted in residual bleach scent lingering on disinfected respirators^6^. Autoclaving respirators have also been investigated, but it is currently unclear if steam decontamination can reliably disinfect respirators without damaging respirator material^7^. Hydrogen peroxide has shown promise as a disinfection method, but additional safety measures must be taken into consideration to properly manage the production of vaporized hydrogen peroxide^8^. The utilization of chemical cleaners for respirator disinfection must be carefully considered as they may change the surface charge of the electrostatically active respirator material or leave behind a chemical residue.

**Figure 1:**
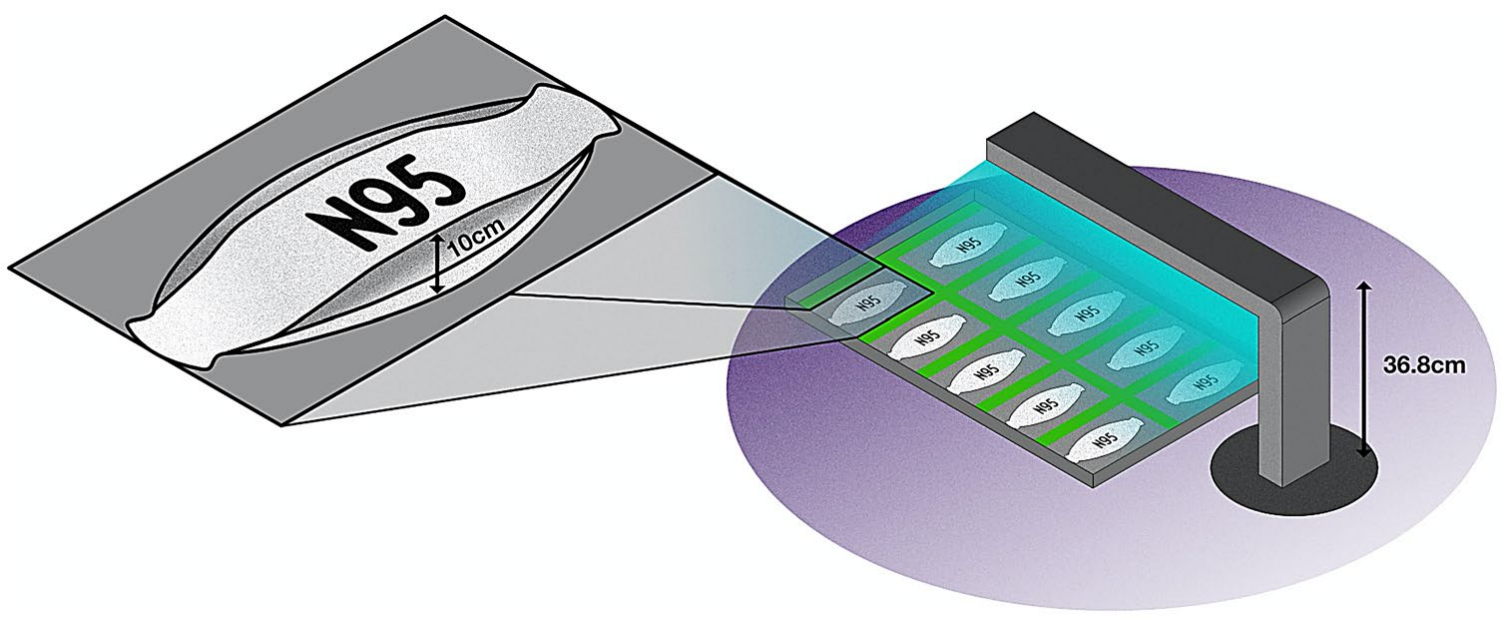
Full-scale setup of N95 respirators as arranged beneath a single arm of the UV light source. The maximum respirator height was measured to be 10 cm. The top of the respirator closest to the light source receives 1142 mJ cm^−2^, and the bottom of the respirator farthest from the light source receives 1000 mJ cm^−2^.

Disinfection techniques that can damage the electrostatic charge and/or the overall structure of N95 FFR material can also significantly impact FFR integrity and fit. FFR integrity is primarily measured using filtration efficiency (i.e. % of particles filtered out by FFR; for N95 FFRs, must be > 95%) and air pressure drop (the resistance of FFR material to air; a large change in this value would indicate that the structure of the material has undergone a significant change)^9^. Fit testing indicates the presence or absence of an adequate FFR face seal on a user (without an adequate face seal, the FFR is ineffective). As such, it is imperative to ensure that disinfection strategies are both effective and maintain the integrity of N95 FFRs to ensure fit and filtration efficiency are not negatively impacted.

UV disinfection provides an alternative to chemical and thermal cleaning as it requires only a calibrated UV light source and a safety enclosure for sequestering excess UV light when the treatment area is in use. In a systematic review involving 43 different N95 FFR models exposed to UV irradiation, all FFRs maintained NIOSH certification standards following UV treatment^10^. Furthermore, it has been shown that UV exposure does not impact N95 respirator material up to 950,000 mJ cm^−2^ ^11^. This exposure of UV light is 950 times greater than the minimum fluence required for achieving the disinfection of a used N95 respirator (1000 mJ cm^−2^), according to the literature^12–14^. As such, UV disinfection is a promising “touchless technology” for repurposing N95 FFRs due to its ability to inactivate a wide range of pathogens while preserving mask integrity. UV disinfection has several additional benefits: treatment can occur without augmenting ventilation systems, the process does not leave behind harmful chemical residues, and disinfection generally occurs over rapid disinfection cycles^15^.

Literature and preprint material involving the impact of UV radiation on N95 FFR integrity are often inconsistent in their definition of what is meant by a “disinfection cycle.” A disinfection cycle usually refers to a set amount of time that a respirator has been exposed to UV light. However, from a UV perspective, this terminology does not provide enough information, as the duration of exposure to light is arbitrary in terms of UV fluence or fluence. UV fluence accounts for the total amount of UV light that the target has been exposed to, and therefore is a clear and comparable term when discussing approaches that use UV technology for respirator treatment/reuse.

Due to the non-oxidizing nature of UV irradiation, disinfection and reuse of N95 FFRs via UV light present a promising method to help maintain the protection of healthcare professionals while treating patients infected with COVID-19. While recent studies have used FFR material coupons to demonstrate the efficacy of UV-disinfection of FFRs^16^, and the ability of the material to maintain structural and functional integrity after UV irradiation^17^, further testing on *intact* FFRs is required to account for potential variations in UV fluence across the uneven surface of the respirator. It remains unknown whether the level of UV treatment required to effectively disinfect N95 FFRs for reuse significantly impacts and compromises the integrity of intact respirators and respirator straps. The objectives of this study were to (1) determine if UV treatment has any easily observable impact on respirator integrity (through visual and manual inspection of N95 FFRs before and after UV treatment, by analyzing N95 FFR respirator layers using microscopy methods, and determining if UV treatment impacts the tensile loading of elastomeric straps); (2) test the impact of UV treatment on N95 FFR user fit via standardized fit test methods; and (3) test the impact of UV treatment on N95 FFR integrity by evaluating particle penetration and air flow through the respirator.

## Materials and methods

### UV disinfection system

A commercially available UV disinfection system (MoonBeam3, Diversey Holdings Ltd.) was used for all treatments in this study. The unit, acquired by the Nova Scotia Health Authority (NSHA), was initially designed for disinfecting hospital rooms and enclosures. The portable device has three individually adjustable arms, each equipped with a 95 W low-pressure bulb that can be positioned in a wide range of configurations to maximize UV light exposure. Average irradiance was measured at a range of reactor arm heights using a spectroradiometer (Ocean Optics USB4000). UV irradiance was also measured across the area where the respirators were placed to ensure no large variation of lamp brightness was observed (Online Resource ESM Table 1 and ESM Fig. 2).

**Table 1:** Experimental design for integrity test after UV treatment on N95 respirators (9210 and 8110S models).

Respirator model	Designation	Number of UV cycles^a^	Total UV fluence per side (mJ cm^−2^)	Total UV fluence applied (mJ cm^−2^)	Replicates
9210	Fit Test	5	5000–5710	10,000–11,420	2
		10	10,000–11,420	20,000–22,840	2
	Integrity	0	0	0	1
		5	5000–5710	10,000–11,420	1
		10	10,000–11,420	20,000–22,840	1
	Air Flow	0	0	0	3
		5	5000–5710	10,000–11,420	3
		10	10,000–11,420	20,000–22,840	3
	Strap tensile loading test	0	0	0	4
		5	5000	10,000	6
		10	10,000	20,000	6
8110S	Fit Test	5	5000–5710	10,000–11,420	2
		10	10,000–11,420	20,000–22,840	2
	Integrity	0	0	0	1
		5	5000–5710	10,000–11,420	1
		10	10,000–11,420	20,000–22,840	1
	Air Flow	0	0	0	3
		5	5000–5710	10,000–11,420	3
		10	10,000–11,420	20,000–22,840	3
	Strap tensile loading test	0	0	0	4
		5	5000	10,000	3
		10	10,000	20,000	3

^**a**^Each UV cycle consisted of a 180-s treatment duration per side of the respirator. In treating each side of the respirator, a full UV cycle (360 s) delivered a fluence of 1000 and 1142 mJ cm^−2^ to the farthest and closest point on the respirator to the light source, respectively. Over five cycles, the total fluence applied to each respirator was 10,000 mJ cm^−2^ (for the region of the respirator farthest from the light source) and 11,420 mJ cm^−2^ (for the area of the respirator closest to the light source). Similarly, 10 UV cycles delivered a total fluence for each FFR of 20,000 to 22,840 mJ cm^−2^.

**Figure 2:**
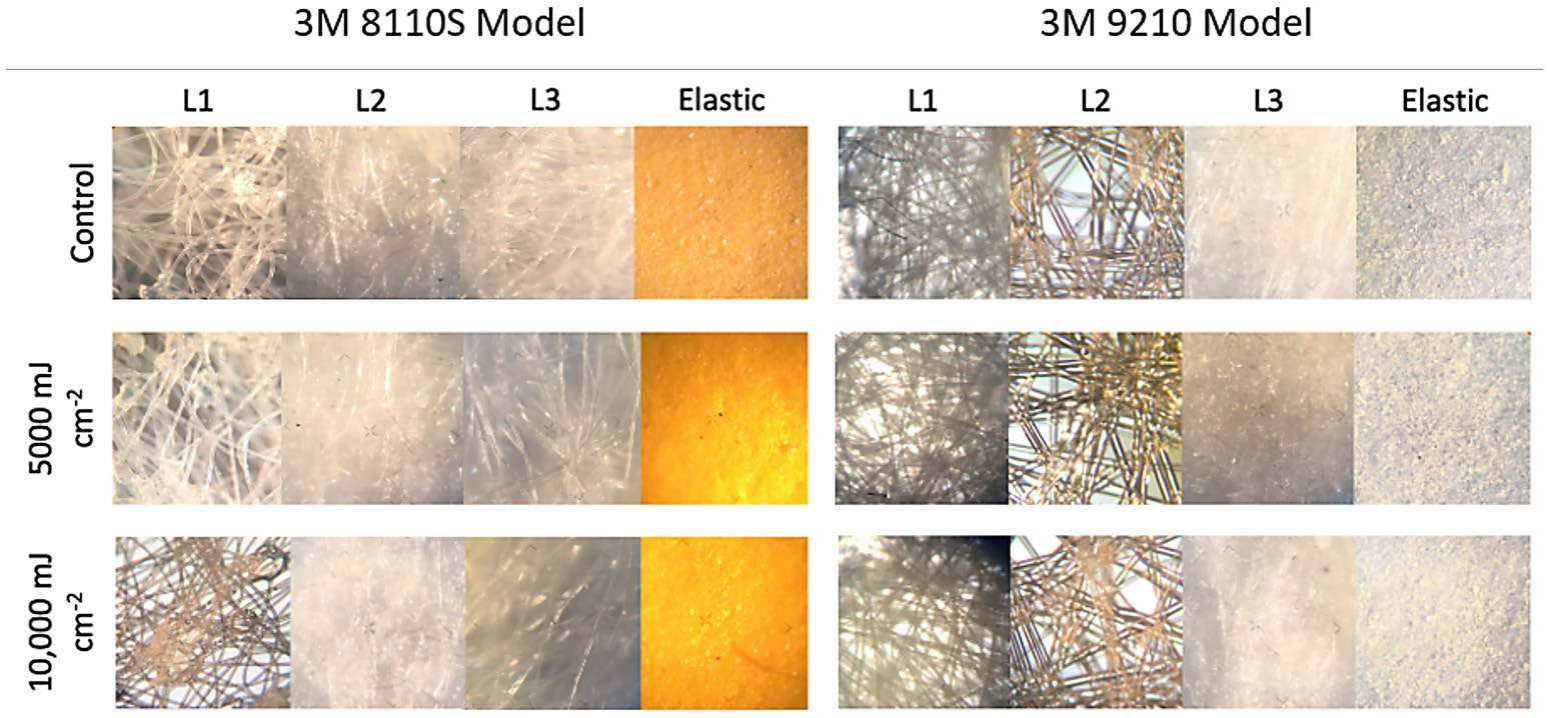
10 × magnification of individual respirator straps and respirator layers for 8110S and 9210 treated and control respirators as viewed under a compound microscope following 0, 5000 and 10,000 mJ cm^−2^ exposure to UV-C light.

A target fluence of 1000 mJ cm^−2^ was selected as the UV fluence per disinfection cycle as, according to present literature, 1000 mJ cm^−2^ per side is the minimum fluence required to disinfect N95 respirators for reuse purposes^13,16,18,19^ effectively. This UV fluence of 1000 mJ cm^−2^, calculated following methods outlined by Bolton and Linden^18^, was obtained at a reactor arm height of 36.8 cm (distance between the bottom surface of the N95 respirator and UV lamp) based on an average recorded intensity of 55.56 W m^−2^ at this height and a UV cycle duration of 180 s.

In previous work, Ontiveros et al*.*^16^ N95 respirator material blocks more than 99% of UV light^16^. This means that for complete disinfection, an application of at least 1000 mJ cm^−2^ *per side* of the respirator is required. For this reason, a UV disinfection cycle was considered complete when both sides of the respirators were exposed to UV light, resulting in a total UV treatment duration of 360 s (180 s per side). Another point that may be overlooked when calculating UV fluence for the disinfection of irregularly shaped objects (such as N95 respirators) is the difference in height across the object, as fluence varies with distance from the UV source.

In this study, reported fluences are measured from the lowest point on an unfolded respirator to ensure that the area farthest from the light source was receiving at least 1000 mJ cm^−2^ of light per cycle. In addition, UV fluence was calculated for the point on the respirator that is nearest to the light source, which reflects an increase in UV fluence of about 140 mJ cm^2^ per cycle from the bottom to the top of the respirator (10 cm) (Fig. 1). Therefore, each N95 respirator received a UV fluence of 1000 mJ cm^−2^ at the bottom of the respirator and 1142 mJ cm^−2^ at the top of the respirator during each 180-s UV cycle. Moreover, the position of each respirator was randomized after each complete cycle to ensure that the spatial position of the respirator did not influence the overall exposure of light received.

### Testing respirator integrity following UV treatment

All N95 respirators used in this study were provided by NSHA. The following parameters to test respirator integrity were assessed: material integrity, respirator fit, particle penetration and airflow through the respirator, and strap tensile loading strength. For this assessment, N95 respirator models (3 M 9210 and 8110S) were exposed to either five or ten 180-s UV cycle lengths on both the top and underside of respirators or straps using the UV disinfection unit, and compared to new and untreated samples, to determine whether N95 respirator integrity is affected by UV light exposure.

N95 FFRs are an essential form of PPE manufactured to protect workers from respiratory hazards rather than for research. Due to the global shortage of N95 FFRs during the COVID-19 pandemic, replicates for each experiment were chosen strategically. Duplicate UV-treated samples of each FFR model were used for the fit test because it is a qualitative rather than quantitative test; in this case, a qualitative outcome, rather than a statistically significant conclusion that requires multiple replicates, was acquired. In addition, it was determined that neither control nor untreated samples were required for this test because it was anticipated that untreated samples would result in a passed fit test. Similarly, only one respirator per model and treatment group was assigned for the integrity test, due to the qualitative non-standardized method and the subjective nature of visual and tactile experiments. Nonetheless, the data was included to assess whether a high dose of UV radiation has a visual or tactile impact on an exposed respirator. To provide stronger conclusions for airflow and tensile tests on respirator straps, which are principal tests in demonstrating the maintenance of respirator integrity following disinfection, larger numbers of replicates were designated for these experiments. The study design for this series of experiments is described in Table 1.

### Overall material integrity

Individual respirators and straps were visually examined to identify whether UV treatment caused visual deformities in the 9210 and 8110S N95 respirators (Online Resource ESM Table 2). The mask material, foam, and metal material (i.e. nose piece and staples) on each treated mask were visually compared to control masks to determine if UV treated had any visual impact on respirators using the naked eye. Control and treated respirator material were also cut into 2-cm diameter circular coupons and separated into their three sublayers (layer 1 (L1), layer 2 (L2) and layer 3 (L3)), with L1 representing the outermost, cloth-like layer of the respirator, L2 representing the more rigid, middle layer of the respirator and L3 representing the thicker, innermost layer of the respirator (closest to the user). Control and treated respirator straps were cut into 1 cm sections, to be further analyzed using a compound microscope under 10 × magnification.

### Tensile loading tests of respirator straps

This experiment identified the effects of UV radiation on the tensile breaking strength of new and intact N95 9210 and 8110S respirator straps when exposed to various UV treatment cycles. Both N95 respirator models used in this study had rubber-like straps. According to manufacturer’s information, the N95 8110S strap material is polyisoprene^19^ and the N95 9210 strap material is braided polyisoprene^20^. To assess the approximate maximum load the respirator straps can withstand with and without UV treatment, tensile tests were performed by adding weight (in approximately 100 g increments) to the straps while they were suspended from a platform to subject the straps to tension. The platform used for this type of test was elevated 2 m from the ground and the straps were loaded until they broke. In addition, serrated clamps held the top end of the strap on the platform to keep the straps in place, and an additional clamp was used at the bottom end to interconnect the strap with the added loads. Respirator straps were detached from the N95 respirators and cut to approximately 18 cm in length and were randomly selected for UV exposure of either 5000 mJ cm^−2^ or 10,000 mJ cm^−2^ on each face of the straps. Straps that were not exposed to any UV light were used as controls.

Engineering stress is defined as *s* = *F/A*_*o*_*,* where *F* is the tensile force applied (load), and *A*_*o*_ is the initial cross-sectional area. Given that the initial cross-sectional area is a parameter dependent on the respirator strap model, and the tensile stress experienced by the straps would depend on the specific elasticity material properties from each strap model, respirators straps were analyzed by grouping them by their corresponding respirator model as with previous studies^11,21^. The cross-sectional areas for the N95 8110S and 9210 respirator straps used in these experiments were 2.5 10^–6^ and 3.0 10^–6^ m^2^, respectively.

Data across all conditions tested for each respirator strap model were compared using a one-way ANOVA at 95% confidence to determine any significant difference in breaking loads between treatment and control groups for each respirator strap model. All statistical analyses were performed using Minitab Express (MINITAB Inc., State College, PA, USA).

### Respirator airflow testing

A set of 18 unused N95 respirators (nine each of 9210 and 8110S models) were used to test the impact of UV exposure on respirator material. Intact respirators were placed under a single arm of the UV disinfection unit (to simulate the full-scale arrangement of disinfecting respirators) and exposed in triplicate independent samples to either 5000 or 10,000 mJ cm^−2^ per side. UV treated respirators were collected and sent to the Health and Environments Research Centre (HERC) Laboratory for filtration performance testing.

Coupons of material (25 mm^2^) were cut from the intact respirators and placed in filter holders within the testing chamber. Filtration efficiency was measured at two time points (5 and 60 min) at an average humidity of 20 ± 1% over a range of particle diameter sizes (20.2 to 445.1 nm), which were measured using a scanning mobility particle sizer (SMPS, TSI 3080). An MKS 223 BD Manometer was used to measure pressure drop at a flow rate of 3 L min^−1^. Mean percent filtration efficiency (with standard deviation) was calculated for each FFR model and UV treatment group across the entire range of particle diameter sizes. A one-way ANOVA at 95% confidence was performed to assess the significant difference in average filtration efficiency between treatment and control groups for each respirator model. Statistical analyses were performed using Minitab 2019 (MINITAB Inc., State College, PA, USA).

### Respirator fit testing

NSHA uses a quantitative method for fit-testing N95 respirators to ensure respiratory protection for healthcare workers. This method involves attaching punctured N95 respirators to a device that measures the user’s seal on each respirator. As N95 respirator models are unique, each one needs to be independently fit tested. Test parameters are measured using a TSI PortaCount 8030 or 8038 in accordance with the CSA Z.94.4–11(R2016), the standard that outlines the requirements for conducting a fit test^22^. In this study, fit testing was performed in duplicate independent respirator samples exposed to 10,000 to 11,420 or 20,000 to 22,840 mJ cm^−2^, as outlined in Table 1.

Each fit test participant completed a test process comprised of seven parameters: (1) normal breathing; (2) deep breathing; (3) head side to side; (4) head up and down; (5) talking out loud; (6) bending at the waist; (7) and back to normal breathing again. Each parameter test was performed for a minimum of 30 s. After each fit test, the PortaCount device displayed a pass/fail outcome based on a numeric value called the “fit factor” (a ratio of particles outside the respirator and particles that have leaked into the respirator)^22^. A resulting fit factor value between 0 and 200 + was displayed for each parameter. An overall fit factor—based on individual fit factors for each test parameter—determined a pass or fail result. The minimum pass fit factor value was 100. Since the overall fit factor depends on the values of each test parameter, the 200 + limit ensured the validity of a total test pass by eliminating the possibility a user achieves a pass with poor numbers on some parameters of their fit test.

NSHA staff in their professional capacity were responsible for the recruitment of the individuals to perform the fit test as part of their occupational health lab. In light of this professional arrangement, NSHA did not seek research ethics revision, which aligns with the Article 2.1 of the Tri-Council Policy Statement (TCPS) (1), "In some cases, research may involve interaction with individuals who are not themselves the focus of the research, in order to obtain information" and "such individuals are not considered participants for the purposes of this Policy. This is distinct from situations where individuals are considered participants because they are themselves the focus of the research". Furthermore, the information obtained from this test came back fully anonymized, which according to Article 2.4 is exempt from REB review.

## Results and discussion

### Overall material integrity following UV treatment

No visual abnormalities (viewed with the naked eye or at 10 × magnification) were observed after 9210 and 8110S respirators had undergone treatment (Table S2) using 5000–5710 or 10,000–11,420 mJ cm^−2^ UV fluence per side (Fig. 2). However, it is difficult to determine the true integral impact of UV treatment on the mask material without using more advanced microscopy methods. Previous work has indicated that UV doses up to 950,000 mJ cm^−2^ (950 × higher than the present study) at 254 nm do not impact the efficacy, structure or integrity of N95 FFRs, indicating that UV treatment may be a promising disinfectant technology for repurposing N95 FFRs^17^. Similarly, another recent study analyzed the effect on respirator material morphology and hydrophobicity after exposure of up to 10,000 mJ cm^−2^ UV fluence using Fourier transform infrared spectroscopy (FTIR), scanning electron microscopy (SEM), optical microscopy, and surface contact angle measurements and found no changes in these properties^17^.

### Tensile loading tests of elastomeric straps

**Figure 3:**
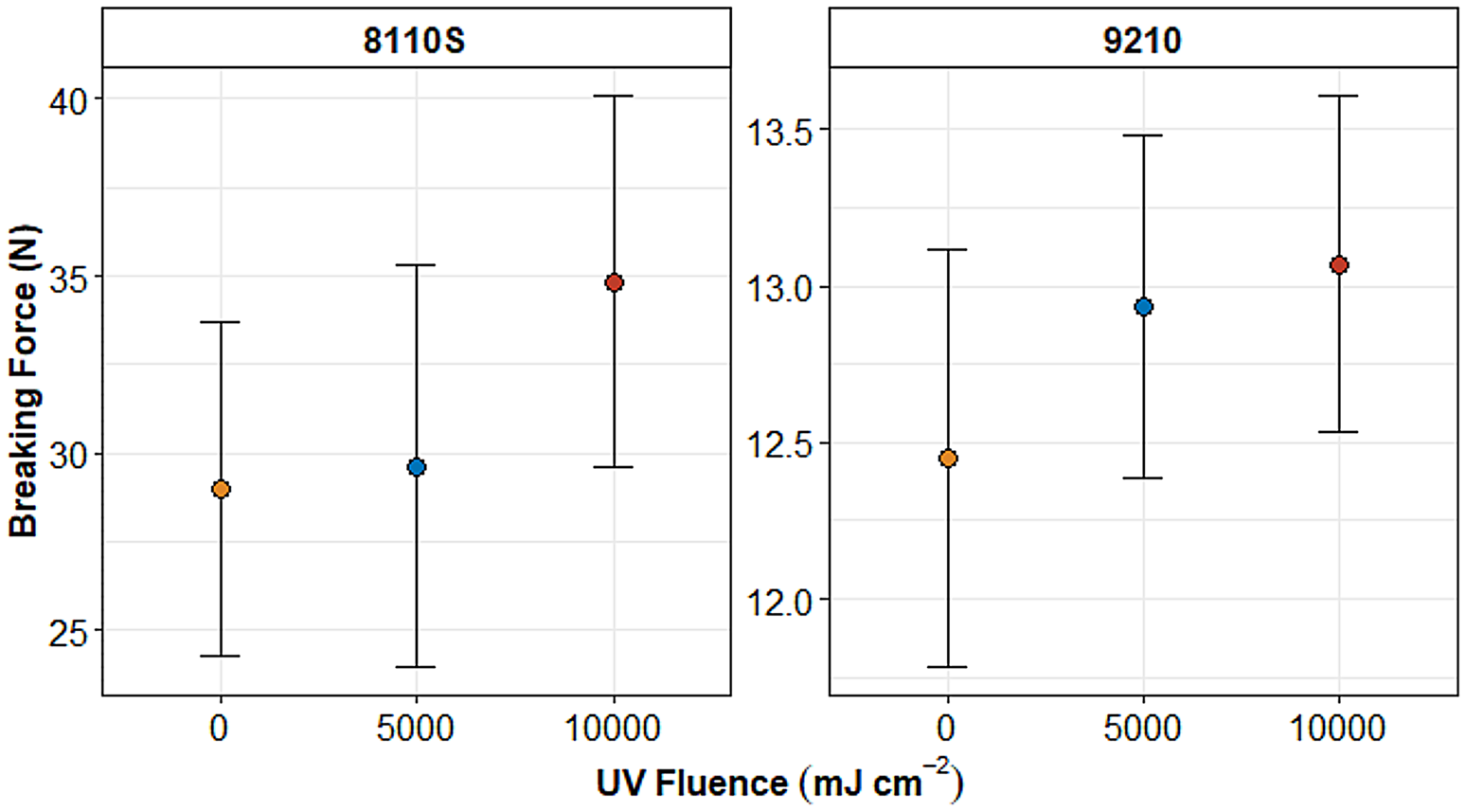
Breaking force vs UV fluence from 8110S and 9210 N95 respirator straps. Error bars represent one standard deviation.

The tensile tests on N95 8110S respirator straps resulted in a mean breaking force of 29.0 ± 4.73, 29.6 ± 5.66, and 34.8 ± 5.23 N from UV treatment of 0 mJ cm^−2^, 5000 mJ cm^−2^, and 10,000 mJ cm^−2^ per side of straps, respectively. Additionally, the N95 9210 respirator straps experienced a mean breaking force of 12.4 ± 0.67, 12.9 ± 0.55, and 13.1 ± 0.53 N, from UV treatment of 0 mJ cm^−2^, 5000 mJ cm^−2^, and 10,000 mJ cm^−2^ on each side of the straps. The breaking loads recorded on the 8110 s respirator straps were approximately two times greater than the 9210-strap model. The results of these tests are summarized in Fig. 3. A complete summary of tensile breaking test results is described in Online Resource ESM Table 3.

A one-way ANOVA with multiple comparisons (null hypothesis: all means are equal) on each respirator strap model, confirm that there were no significant differences in the mean breaking loads from the N95 8110 s respirator straps exposed to UV fluences (5000, and 10,000 mJ cm^–2^ per side) and the controls (p-value 0.348, α = 0.05). Similarly, using the same one-way ANOVA test on the N95 9210 respirator straps showed no significant difference in the mean breaking load when each face of the straps was exposed to 0, 5000, and 10,000 mJ cm^–2^ UV fluences (p-value 0.264, α = 0.05). Given that the data reported form this tensile test indicated no significant difference in mean breaking loads for straps exposed to various UV disinfection fluences within their corresponding respirator model groups, UV fluences up to the tested 10,000 mJ cm^−2^ did not appear to have a significant impact on the breaking loads for the respirator straps tested.

Lindsley et al*.*^11^ investigated the effect of UV exposure on respirator straps at fluences of 590,000, 1,180,000, and 2,360,000 mJ cm^−2^. However, the average breaking load for 9210 respirator strap controls (0 mJ cm^−2^) was 15 N, approximately 2.6 N (0.26 g weight load) higher than the results shown in this study for the same respirator model. At a UV fluence of 590,000 J cm^−2^ (590 times higher than the UV fluence applied in this study), Lindsley et al.^11^ reported a 10 to 21% decrease in breaking strength for the 9210-respirator strap model. In addition, the authors suggested that the strength of the respirator straps is less affected by UV fluence than the strength of the respirator material itself. Moreover, Heimbuch and Harnish^21^ evaluated the effects 1800 mJ cm^−2^ on 15 FFR respirator strap models (none of which were tested in this study). No effect on the strength of the respirator straps was reported at that fluence. Similarly, Zhao et al.^17^ found no change in tensile strength on the respirator elastic after a UV fluence of 10,000 mJ cm^−2^ was applied. Although there are some variations in applied fluences and types of respirator models tested in the literature, the results of this study are consistent with the findings reported in these previous studies.

Additionally, it is important to highlight the limitations of this specific study. A quick and simple tensile loading test using weights was performed to identify differences in breaking loads after and before UV treatment. Higher specialized equipment, appropriate to the material strength properties of these respirator straps, such as a universal testing machine for tension while following the applicable ASTM standard is recommended to verify these results. While only two models of N95 respirator straps were tested due to limited FFR availability, other respirator models would need to be tested to confirm the non-significant effect of UV fluences up to 10,000 mJ cm^–2^ on respirator strap breaking strength. However, the literature indicates that a significantly higher fluence (590,000 mJ cm^2^) would be required to reduce the strap breaking strength by an average of 14% across four models of respirators^11^ and therefore reduce the fit performance of the FFRs.

### Airflow test on UV-treated respirators

**Figure 4:**
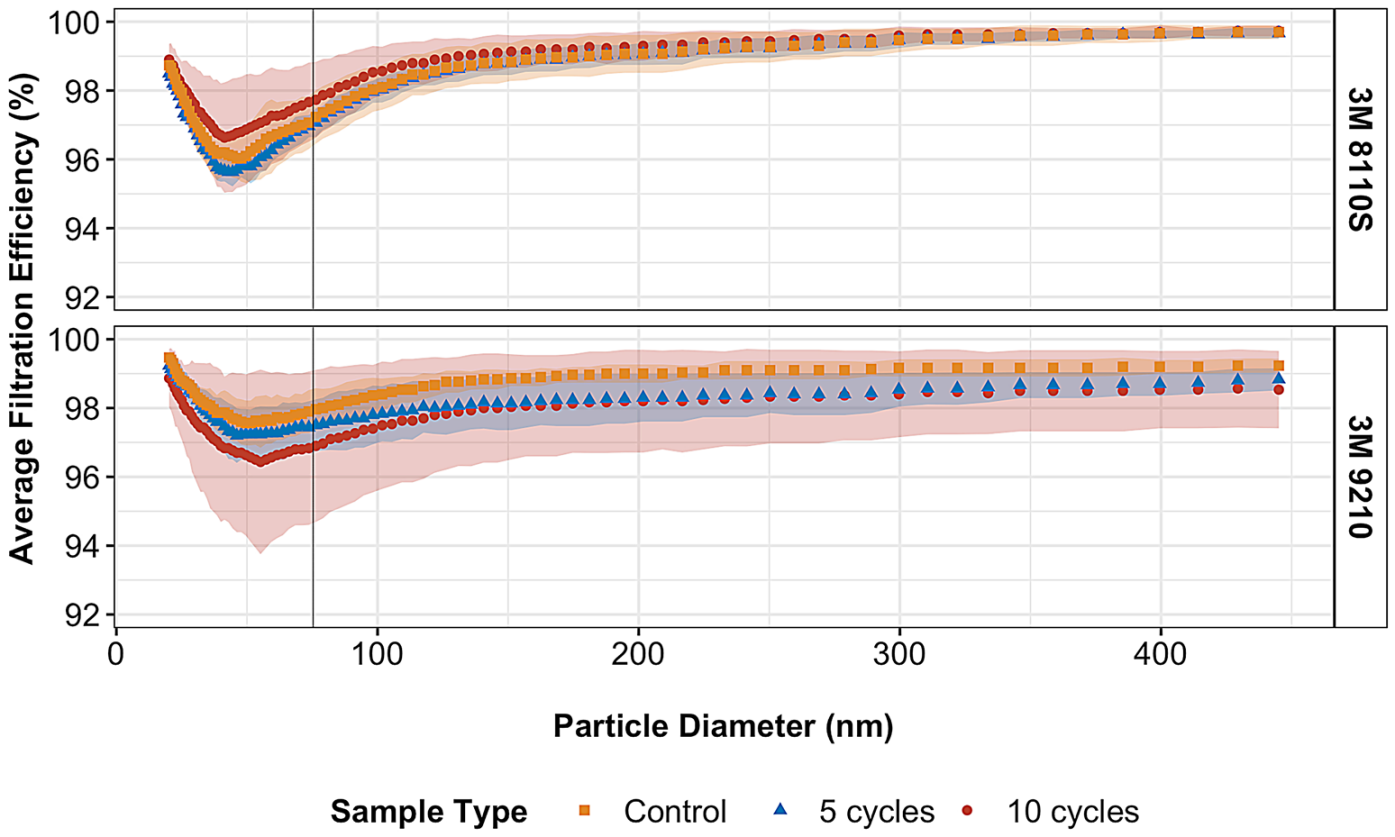
Filtration efficiency of airflow testing in 8110S and 9210 N95 respirators models compared to particle diameter. 75 nm count median diameter is noted by the dashed line. The shaded area represents standard deviation, n = 3. Software used: R studio, 1.3.959. Libraries: readxl, tidyverse, reshape2, ggthemes, ggsci, RColorBrewer.

The airflow test results indicate that UV exposure alone does not impact the filtration performance of N95 respirators. The average filtration efficiency did not fall below 95% for any of the respirator types or UV fluence levels (Fig. 4,^23^). For both 8110 and 9210 respirator models, a one-way ANOVA with UV treatment as a factor and average filtration efficiency across all particle diameters as a response indicating that the untreated samples and samples treated with 5000 and 10,000 mJ cm^−2^ per side were not significantly different (*p* value > 0.05). Therefore, respirators exposed to up to 10 treatment cycles (10,000 mJ cm^−2^ per side) maintained a 95% filtration capacity, which successfully meets requirements for NIOSH compliance^24^. Furthermore, NIOSH certification requires a minimum collection efficiency of 95% when tested with non-oil particles (e.g., NaCl) with a count median diameter (CMD) of 75 ± 20 nm, a geometric standard deviation (GSD) of < 1.86, and a mass median aerodynamic diameter (MMAD) of ~ 300 nm^25^. The most penetrating particle size (MPPS), which represents the optimal particle diameter for measuring filter performance^23^, for most N95 FFRs ranges from 30 to 100 nm^25^. The results of this test show that when challenged at this particle size, all FFRs in each exposure group demonstrated a high level of performance (average filtration efficiency > 96%).

Furthermore, the initial pressure drop in data for 8110S and 9210 N95 FFR models is presented in Online Resource ESM Table 4. There was no statistically significant difference in the initial pressure drop between the UV treated samples with 5000 and 10,000 mJ cm^−2^ per side and untreated samples. Repeated, fluence-based exposure of respirator material to UV light does not degrade filtration performance. These results were consistent with previous studies^17^ that found no significant difference in filtration efficiency or pressure drop after up to 10,000 mJ cm^−2^ of UV radiation on respirator coupons. A more recent study that also evaluated filtration performance of N95 respirators up to 1488 mJ cm^−2^ found that UV radiation does not affect filtration efficiency in UV treated respirators^26^. Thus, UV exposure alone does not contribute to respirators’ degradation in a meaningful way and other factors may need to be considered in order to understand the implications of respirator reuse.

### Respirator fit testing on treated respirators

After evaluating several different fit factors, results from standardized fit testing indicated that a total UV fluence of 10,000 to 11,420 or 20,000 to 22,840 mJ cm^−2^ did not impact respirator fit (Online Resource ESM Table 1). All control and treated respirators received a fit factor value of > 100, achieving an overall passing score based on all criteria tested. These results indicate that N95 respirators can be successfully disinfected up to ten times without risk to user fit and overall integrity.

In a recent study that quantified the ability of N95 FFR material (4 × 4 cm coupons of two models, 3 M 1860 and Moldex 1500) to remove virus-sized aerosol particles, and subsequently characterized impacts of 254 and 265 nm UV-C irradiation exposure on the materials, no adverse effects were observed on the materials’ ability to remove aerosolized virus-sized particles^17^. Furthermore, there was no apparent change in the polymer structure, morphology, or surface hydrophobicity for FFR material layers and no change in pressure drop or tensile strength. These results are consistent with those reported in this study and provide additional evidence that UV disinfection of N95 FFRs is a viable option for safe and effective repurposing of these essential devices amid global shortages experienced during a pandemic.

## Conclusions

The present study aimed to evaluate the impact of UV irradiation (five and 10 UV cycles, corresponding to UV fluences of a minimum of 5000 and 10,000 mJ cm^−2^, respectively, per side of respirator) on the overall fit, filtration efficiency and structural integrity of 8110S and 9210 N95 respirators. Standardized fit tests, airflow filtration tests, visual inspections and tensile loading tests were applied. Visually, there seemed to be no significant impact on N95 respirators exposed to UV fluences of 10,000 mJ cm^−2^ per side of the respirator; however, further testing is needed to determine if this is definitively the case. Similarly, there were no significant differences between the breaking strength of the respirator straps before and after UV irradiation using fluences of up to 10,000 mJ cm^−2^. Moreover, both respirator models passed a qualitative fit test after UV irradiation using UV fluences of 5000 and 10,000 mJ cm^−2^. There were no significant differences between control and UV treated samples for the airflow filtration test. Both models maintained an average filtration efficiency above 95%, which indicates that UV irradiation up to a fluence of 10,000 mJ cm^−2^ per side of respirator does not impact respirator integrity. Overall, the UV fluences that were tested in the present study did not significantly impact the breaking strength of respirator straps, nor the fit and airflow filtration capacity of these two N95 respirator models that were evaluated. These results indicate that N95 respirators may be able to be disinfected and reused up to ten times without significant impact to mask efficiency. This work will help to build the discussion around using UV technology for the decontamination of N95 respirators for reuse in case of potential mask shortages during pandemic events.

This is the first study investigating the impact of UV-disinfection on intact N95 FFRs, which accounts for the changes in UV fluence across the uneven surfaces of the respirators. One notable limitation of this study is the low number of replicates for some of the experiments. Due to the global shortage of N95 FFRs during the COVID-19 pandemic, the authors strategically selected numbers of replicates for the tested parameters. Future work may involve collaborations between researchers and hospitals to provide ongoing quality assurance testing of used and disinfected FFRs as they become available.

## Supplementary Information

Supplementary Information.

## Data Availability

The data used in this manuscript has been included in the Electronic Supplementary Material section.
