# Supplementary material for: Assessing the impact of multiple ultraviolet disinfection cycles on N95 filtering facepiece respirator integrity
Source: Sci Rep. 2021 Jun 10;11:12279. doi: 10.1038/s41598-021-91706-1 (PMC8192506; doi:10.1038/s41598-021-91706-1)
Supplement: Supplementary file 1 — Supplementary Information. [file 41598_2021_91706_MOESM1_ESM.pdf]

SUPPLEMENTARY MATERIAL

Title

Assessing the impact of multiple ultraviolet disinfection cycles on N95 filtering facepiece respirator integrity

Authors

C. Carolina Ontiveros[1] M.A.Sc, Crystal L. Sweeney[1] PhD, Christopher Smith[2] P.Eng, BRM, Sean MacIsaac[1] B.Eng, Jessica L. Bennett[1] B.Sc, Sebastian Munoz[1] M.A.Sc, Amina K. Stoddart[1] PhD, P.Eng and Graham A. Gagnon[1] PhD, P.Eng †

ESM Table 1 Relative Intensity of UV light source across the total area of the illuminated area (n=3). Positional indices correspond with **ESM Fig. 2**

Position	Relative Intensity	Standard Deviation
P1	66%	3%
P2	68%	4%
P3	108%	1%
P4	134%	14%
P5	124%	6%

ESM Table 2 Manual inspection of N95 respirators before and after UV treatment

FFR Model	UV Treatment per side (mJ cm^{-2})	Overall Look	Overall Feel	Straps
8110S	0	Respirator is stark white, straps are mustard yellow, text is light grey, no visual abnormalities in respirator material, staples, or foam. Some small visual defects in strap and metal nose piece.	Respirator material is taut, stiff, and thick near metal nose piece, thinner and more flexible near bottom of respirator. Straps are fastened securely by staples. Foam is light and spongy. Respirator is easily compressed horizontally but not vertically.	Strap is strong and flexible, fastened securely to respirator material. Some small vertical marks on the inside strap near the staple on the top left side of respirator.
	5,000 to 5,710	Permanent marker ID appears faded on front and underside of respirator. No other visual differences from the control.	No tactile differences from control	No visual differences from control, but feels less flexible
	10,000 to 11,420	Permanent marker ID appears faded on front and underside of respirator. No other visual differences from the control.	No tactile differences from control	No visual differences from control, but feels less flexible
9210	0	Respirator is stark white, straps are light blue, text on front and underside of respirator is light grey. Sponge on nose piece is darker, less porous and more durable than 8110S model. No visible abnormalities in any respirator material	Respirator is easily compressible vertically and horizontally. Internal metal nose piece is very flexible in comparison to 8110S model. Upper and lower respirator material is more flexible than main respirator material (i.e. material covering mouth).	Strap is flexible, not as strong as the 8110S models. Fastened securely to respirator material
	5,000 to 5,710	Permanent marker ID appears slightly faded on front and underside of respirator. No other visual differences from the control.	No tactile differences from control	No visual or tactile difference from control
	10,000 to 11,420	Permanent marker ID appears slightly faded on front of respirator. No other visual differences from the control.	No tactile differences from control	No visual or tactile difference from control

ESM Table 3 Mean applied breaking force and plastic deformation following UV treatment of respirator straps

Respirator Model	UV Treatment per side (mJ cm^{-2})	n	Applied Breaking Force (N)		Breaking Strength (KPa)	
			Mean	Standard Deviation	Mean	Standard Deviation
8110S	0	4	29.0	4.73	12096.5	1893.1
	5,000	3	29.6	5.66	13158.0	2266.0
	10,000	3	34.8	5.23	13153.2	2092.4
9210	0	4	12.4	0.67	4143.6	222.9
	5,000	6	12.9	0.55	4428.7	182.8
	10,000	6	13.1	0.53	4429.2	178.1

ESM Table 4 Results from air flow testing on two N95 respirator models after UV treatment (n=3)

N95 FFR Model	Sample Type	UV Fluence per side (mJ cm^{-2})[a]	Replicate #	Average filtration efficiency across particle diameter (%)	Initial pressure drop (Pa)[b]
8110	Untreated	0	1	97.96	200
			2	97.56	137
			3	98.65	121
	UV treated	5,000 to 5,710	1	98.13	184
			2	97.59	188
			3	98.00	95
		10,000 to 11,420	1	97.62	145
			2	99.12	153
			3	98.50	96
9210	Untreated	0	1	98.15	177
			2	98.72	84
			3	98.81	164
	UV treated	5,000 to 5,710	1	98.69	188
			2	98.09	121
			3	97.48	144
		10,000 to 11,420	1	98.34	193
			2	95.81	149
			3	99.00	143

[a]Applied fluence per side of respirator
[b]Pressure Drop (Pa) measurement: MKS 223 BD Manometer, @ 25 mm filter (5 cm^2) 3 LPM. These are unofficial ratings.

ESM Table 5 Fit test results for N95 respirator models 9210 and 8110S after receiving UV fluences of 10,000 to 11,420 mJ cm^{-2} (5,000 to 5,710 mJ cm^{-2} per side) or 20,000 to 22,840 mJ cm^{-2} (10,000 to 11,420 mJ cm^{-2} per side) (n=2)

N95 FFR Model	# of replicates	UV Fluence per side (mJ cm^{-2})[a]	Overall Fit Factor	Pass/Fail[b]
9210	2	5,000 to 5,710	200	Pass
	2	10,000 to 11,420	200	Pass
8110S	2	5,000 to 5,710	185	Pass
	2	10,000 to 11,420	185	Pass

[a]Applied fluence per side of respirator

[b]Parameters tested include normal breathing (initial), deep breathing, head side to side, head up and down, talking out loud, bending over, and normal breathing (final)

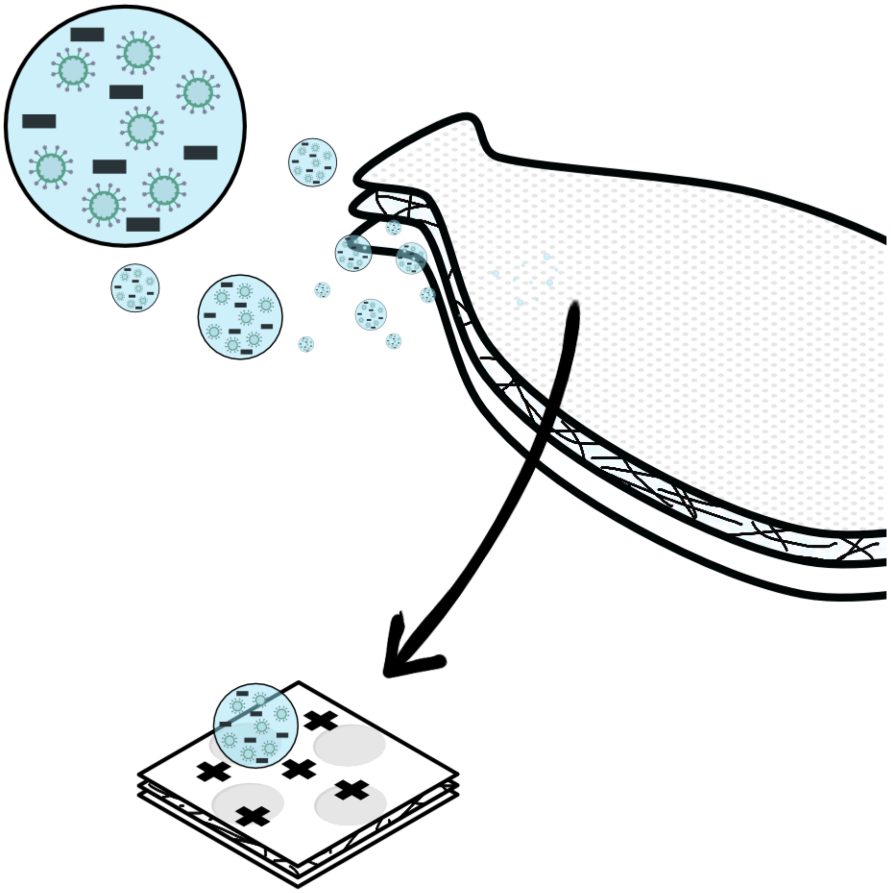

ESM Fig. 1 Electrostatic filtration mechanism for N95 respirators

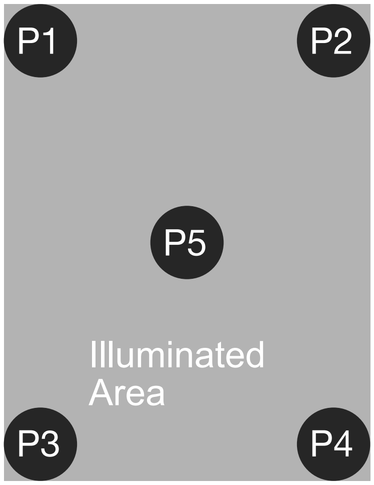

ESM Fig. 2 Spectroradiometer position for relative intensity measurements
